# Ultrasound Measurements of Fetal Thyroid: Reference Ranges from a Cohort of Low-Risk Pregnant Women

**DOI:** 10.1155/2019/9524378

**Published:** 2019-12-17

**Authors:** R. M. Barbosa, K. C. Andrade, C. Silveira, C. M. Almeida, R. T. Souza, P. F Oliveira, Jose Guilherme Cecatti

**Affiliations:** ^1^Department of Gynecology and Obstetrics, University of Campinas, School of Medical Sciences, Campinas, Brazil; ^2^Statistics Unit, University of Campinas, School of Medical Sciences, Campinas, Brazil

## Abstract

**Background:**

Adequate thyroid function is essential for normal growth and development of the fetus. Sonographic recognition of alterations in fetal thyroid dimensions may be the first sign of thyroid dysfunction, permitting early diagnosis and intervention. The main goal of this study was to build curves with reference values for ultrasound measurements of the fetal thyroid from 14 to 40 weeks of gestation.

**Methods:**

This is a prospective longitudinal study of 90 Brazilian pregnant women, complementary to a cohort multicentre study named “WHO multicentre study for the development of growth standards from fetal life to childhood: the fetal component.” Pregnant women without any pre-existing conditions that might affect fetal growth received antenatal care from the first trimester until childbirth, undergoing serial ultrasound evaluations of the fetus, including the thyroid. Longitudinal, anteroposterior, and transverse diameters of both thyroid lobes were measured in the fetus. Fetal thyroid lobe volume was also estimated. By quantile regression analysis, reference curves of measurements were fitted according to the gestational age.

**Results:**

A reference standard of thyroid growth was defined during pregnancy by fitting curves of its measurements. Reference values for the 10^th^, 50^th^, and 90^th^ centiles of fetal thyroid measurements (longitudinal, anteroposterior, transverse diameters, and lobe volume) were defined, from 14 to 40 weeks of gestation.

**Conclusion:**

We provided a reference curve of optimal thyroid development in a low-risk population that can be used as a standard of comparison to diagnose deviations from the norm. In addition, we demonstrated an alternative and simplified method for early recognition of thyroid morphological alterations by an individualized technique to evaluate the thyroid lobes.

## 1. Background

Adequate thyroid function is essential for normal growth and development of the fetus. Thyroid dysfunction is associated with a number of adverse outcomes including intrauterine growth restriction, alterations in amniotic fluid volume, fetal tachycardia, cardiac failure, craniosynostosis, and even fetal death [1–3]. Pregnant women with thyroid disease, essentially cases with positive antithyroid receptor antibodies and users of antithyroid drugs, are at higher risk of developing alterations in fetal thyroid function [4–7].

Hormone measurement by cordocentesis is a definitive method to evaluate fetal thyroid function [8–10]. Nevertheless, such an invasive procedure is not devoid of risks [11, 12], and noninvasive methods should be used for screening. Ultrasound measurements of fetal thyroid dimensions have been shown to be a good parameter to identify fetuses with probable alterations in thyroid function [7, 13, 14]. Small increases in this gland may be the first sign of thyroid dysfunction, permitting early diagnosis and intervention, avoiding severe consequences to the fetus [13, 14].

Although the diagnosis of goiter may be relatively easy, the identification of subtle enlargement in fetal thyroid measurements is much more difficult. Therefore, knowledge of reference thyroid measurements is fundamental. There are few reference curves for fetal thyroid measurements [14–20], derived from studies that used different methods and results. Furthermore, thyroid development and function are dependent on various aspects related to homeostasis of the individual, which may be influenced by diverse socioeconomic, anthropometric, nutritional, and other coexistent conditions. Therefore, homogeneity of the study population is of crucial importance to assess a reference curve for thyroid development [21].

The aim of this study is to construct reference curves of values for ultrasound measurements of the fetal thyroid in its three dimensions and lobe volume, according to sex and side of the thyroid lobe measured, in a pregnant women cohort without any pre-existing conditions that might limit fetal development.

## 2. Methods

This study is complementary to a longitudinal prospective study, based on subjects of a Brazilian centre of a multicentre international prospective study, named *WHO multicentre study for the development of growth standards from fetal life to childhood: the fetal component (Fetal Growth Study)* [22]. This cohort study included low-risk pregnant women in 10 different countries, receiving follow-up care from the first trimester of pregnancy to the time of childbirth. Its aim was to obtain sonographic data related to fetal growth, among other maternal and fetal parameters. Study protocol was previously published [22] and approved by the WHO Research Ethics Review Committee. In addition, approval was also obtained from the local institutional review board of each centre, including Brazil (local IRB letter of approval 406/2008).

Participant selection for the Fetal Growth Study involved health, environmental, and socioeconomic conditions. These conditions had to be considered optimal and not to interfere with fetal growth. Included in the study were pregnant women aged 18 to 40 years, with a BMI of 18 to 30 Kg/m^2^ (allowing inclusion of overweight but not obese pregnant women), singleton pregnancies, gestational age by the date of the last menstrual period between 8 weeks and 12 weeks and 6 days, confirmed by CRL (crown-rump length) measurement, socioeconomic status, and educational level that would not interfere with fetal growth. Exclusion criteria were smoking, current or past evidence of pathological, clinical, gynecological, and obstetric conditions, or socioeconomic, educational, and nutritional conditions that could interfere with fetal growth.

For the current analysis, 90 of the 157 participants in the Brazilian centre of the multicentre study also complementarily underwent serial ultrasound evaluation of fetal thyroid measurements. To estimate the sample size, data from Bernardes et al. [19] reported the mean transverse diameter of 15.1 mm and standard deviation of 1.87 mm to 28 weeks of gestational age. With type I error of 5% and type II error of 20%, the minimum sample size estimated was 100 observations (measurements). In this study, the 90 pregnant participants underwent measurement of 507 thyroid lobes distributed among seven gestational age periods between 14 and 40 weeks of gestation.

Examinations were performed in the Unit of Ultrasonography of the Department of Obstetrics in the Women's Hospital at the University of Campinas, from 2/2013 to 8/2014 by a single sonographer with experience, trained and certified for the multicentre study. The first sonographic evaluation was conducted between 8 weeks and 12 weeks and 6 days. The transvaginal technique was used, and gestational age was confirmed by measuring the CRL. Subsequent visits were scheduled at 4-week intervals and occurred at 14, 18, 24, 28, 32, 36, and 40 weeks. Fetal thyroid measurements were obtained from study participants at the time of ultrasound follow-up.

Only one equipment was used for measurements (Voluson Expert E8, General Electric, Kretz Ultrasound, Zipf, Austria). Both thyroid lobes (right and left) were measured separately in their three diameters (longitudinal, anteroposterior, and transverse), and their volumes were calculated by using the formula for ellipsoid structures: Vol = (π/6) × *L* × AP × *T*, where *L*, AP, and *T* are the longitudinal, anteroposterior, and transverse diameters, respectively.

**Figure 1:**
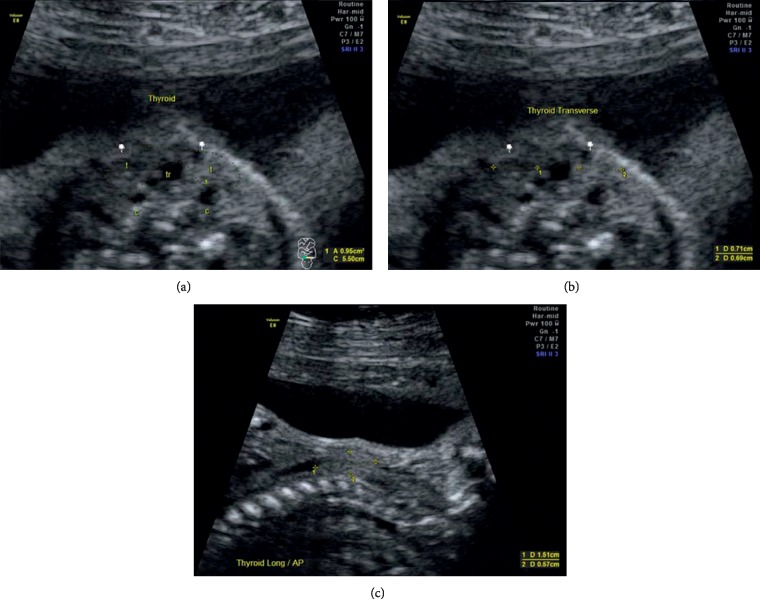
Ultrasound scan showing measurements of fetal thyroid lobe diameters in a 31-week gestation. (a) Transverse cut of the neck showing the thyroid lobes (t) around the trachea (tr) and in front of the carotid arteries (c). (b) Transverse diameters of both lobes of the fetal thyroid. (c) Longitudinal and anteroposterior diameters of the fetal thyroid lobe in the sagittal plane.

Measurements of the fetal thyroid were taken with the fetus preferentially in the posterior dorsal position, although inclinations with corrections up to 90 degrees were accepted. Fetuses in the anterior dorsal position, in which rotation of the transducer was unable to correct this position, presence of nuchal cord, and technical difficulties were reasons for failure to obtain measurements. The fetal thyroid was measured in a transverse view through the fetal neck, and the thyroid was visualized between both carotid arteries, surrounding the trachea. The transverse diameter of each lobe was measured in this plane, placing the calipers on the outer borders of the boundaries of each thyroid lobe. In planes perpendicular to this plane, left and right parasagittal plane of the fetal neck, measurements of the anteroposterior and longitudinal diameters of each thyroid lobe were obtained bilaterally, perpendicular between them (Figure 1). In all measurements, the image was appropriately magnified to occupy around 2/3 of the screen, and calipers were placed from the outer border to the outer border of relevant structures.

For the analysis, initially, the sociodemographic and pregnancy characteristics of the sample of women were described. To describe thyroid lobe measurements according to the side and gender of the fetus, tables of descriptive statistics of these measurements were compiled with values including the means, medians, standard deviations, and minimum and maximum values. To compare measurements between the right and left thyroid lobes, ANOVA test for repeated measures was used. Data were rank transformed because the distribution of measurements was not normal. To compare the means between male and female fetuses, the Mann–Whitney test was used. The volume of each thyroid lobe was estimated by the formula previously specified. This measure was later normalized, using a Box–Cox transformation [23]. By quantile regression analysis, reference curves of thyroid measurements were created, according to gestational age with their respective 10^th^, 50^th^, and 90^th^ centiles. The significance level adopted for this study was 5%.

## 3. Results

Sample characteristics of 90 low-risk pregnant participants in this study are shown in Table 1. Caucasian and nulliparous women constituted the majority of study participants. Of these women, around 40% were 30 to 34 years of age and 34% were overweight. The majority of deliveries occurred at full term (95%). Newborn infants were described as having good vitality, and low birth weight rates occurred in 4.4%.

Follow-up care of the 90 pregnant women resulted in a total of 255 assessments of thyroid measurements, in which 507 thyroid lobes were measured (255 right lobes and 252 left lobes).  Table 2 shows that there was no significant difference in measurements between the right and left thyroid lobes. A comparison of the mean thyroid measurements between male and female fetuses also indicated that there was no significant sex-related difference (Table 2). Therefore, all measurements performed, irrespective of sex or side of the fetus, could be used to construct the curves and define reference percentiles.

**Figure 2:**
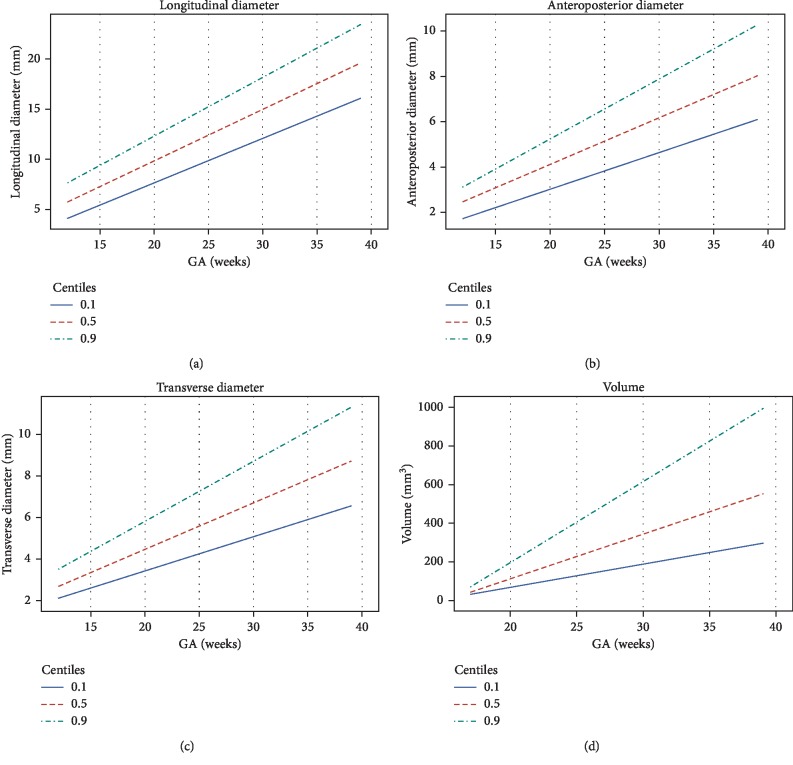
Curve of values for the fitted 10^th^, 50^th^, and 90^th^ centiles of the longitudinal, anteroposterior, and transverse diameters and fetal thyroid lobe volume according to gestational age.

Through quantile regression analyses, reference curves were built for each fetal thyroid measurement according to gestational age (Figure 2). Regression equations were generated for each measurement, as shown in Table 3. The 10^th^, 50^th^, and 90^th^ centiles were defined for each gestational age, and the derived values are shown in Table 4.

## 4. Discussion

In the current study, curves of reference ranges were constructed for diameters and volume of fetal thyroid lobes during pregnancy (from 14 to 40 weeks of gestation). No differences in anatomic topography of the lobe (right or left) or fetal gender were observed. Therefore, each lobe was considered an independent unit of analysis, increasing statistical power of the available sample. In addition, thyroid measurements grew linearly during the course of pregnancy.

One great advantage of the current study is that it is a complementary analysis of an important longitudinal cohort study, in which the study population was rigorously selected and received follow-up care [22]. Diverse health conditions or environmental, nutritional, and socioeconomic relevant factors that might interfere with fetal growth were withdrawn. It is known that thyroid function is associated with iodine ingestion [24, 25]. Therefore, understanding maternal nutritional status would be of the utmost importance when studying the fetal thyroid.

Since this is a prospective longitudinal study, the measurements obtained are useful not only for comparing thyroid size at a specific gestational age but also for the evaluation and conclusion on how thyroid growth and development occurs throughout pregnancy.

There are few publications on ultrasound measurements of the fetal thyroid aimed at exploring reference curves of the gland. These studies have some divergent results that may be attributed to different methods employed, types of study, and measurement techniques used [14–20]. Furthermore, different populations, ethnic and anthropometric factors, as well as iodine ingestion may have contributed to the discrepancy found [20, 24, 25].

The majority of studies evaluated the fetal thyroid in a single plane, with measurements of circumference [14, 16–20], total transverse diameter [14, 16, 18–20], or thyroid area [19, 20]. These measurements ultimately included the trachea, and the influence of its presence on the final thyroid measurement was not taken into account. Furthermore, several studies used an automatic ellipsis to calculate these measurements [16, 17, 20], which often did not correspond exactly to the irregular limits of the fetal thyroid. In our study, the fetal thyroid lobes were measured separately, without including the trachea. As demonstrated, there was no difference between the right and left lobes. This conclusion enables us to infer that measurement of only one fetal thyroid lobe may reflect the size of the whole gland. It can be used as an easier option when screening for thyroid development deviations. In fact, we used the 10^th^ and 90^th^ percentiles of the measurements as the lower and higher limits for identifying possible altered values thinking in a screening process. It should be the object of an additional validation in the future, but probably where any altered value is detected, additional investigation would be recommended to confirm any growth deviation in fetal thyroid.

No other study has sonographically compared the right and left lobes of the fetal thyroid nor compared the difference between thyroids of male and female fetuses. Our results, however, are in agreement with a study on fetal autopsy findings [26]. In our study, there was a slight difference in the number of sides of thyroid lobes evaluated. The lower number of left lobes evaluated was due to technical difficulty and incomplete measurements in some examinations. We recognize that technical difficulty may occur in a few cases. However, the technique has been shown to be reproducible and is easily performed since the number of lost measurements was actually low.

We assessed the fetal thyroid lobe in its three dimensions (longitudinal, anteroposterior, and transverse), which enabled the calculation of its volume. Only one previous study evaluated fetal thyroid volume, regarding it as the best parameter for assessment of thyroid development [15]. Nevertheless, that study created reference curves only for thyroid volume and did not show reference values for each thyroid measurement individually. Furthermore, it used a formula to calculate the total thyroid volume including the isthmus, which was not measured by ultrasound [15].

Although it may seem to be more burdensome, demanding technique and experience from the examiner, sonographic evaluation of the fetal thyroid in its three dimensions may allow for earlier detection of deviations from the norm, particularly volume calculation. In addition to creating a reference curve of thyroid lobe volume, this study also established reference values of independent thyroid measurements (longitudinal, anteroposterior, and transverse), which could facilitate a comparison during routine exams, especially in conditions where it is impossible to perform all or a particular sonographic measurement. Therefore, this study provides new and independent parameters that may contribute to the identification of thyroid growth deviations.

For this study, a possible limitation refers to the intraobserver and interobserver variabilities of diverse measurements of the fetal thyroid that were not assessed. However, in similar studies in which these variabilities were tested, it was demonstrated that the fetal thyroid can be measured accurately with a high level of intraobserver and interobserver concordance, ensuring reproducibility of the method [16, 17, 19, 20].

Although the number of measurements was statistically sufficient to construct reference values, it is likely that an even larger number of measurements at each gestational age could strengthen the impact of the results and narrow down some variations to the same gestational age.

## 5. Conclusion

We have provided a reference curve of fetal thyroid lobe measurements (longitudinal, anteroposterior, transverse diameters, and volume), from 14 to 40 weeks of gestation in a low-risk population, which may be used as a standard of comparison to diagnose deviations from the norm. Furthermore, we demonstrated a simplified and alternative method for early recognition of thyroid alterations, applying a technique of individualized evaluation of the thyroid lobes for diagnosis and treatment of fetal thyroid disorders.

## Figures and Tables

**Table 1:** Characteristics of study participants (*n* = 90).

	*n*	%
*Maternal age (years)*		
18–24	12	13.3
25–29	23	25.6
30–34	36	40.0
≥35	19	21.1

*BMI (Kg/m* ^*2*^)		
Normal (18–24.99)	59	65.6
Overweight (25–29.99)	31	34.4
Ethnicity		
Caucasian	87	96.7
African	3	3.3

*Parity*		
Nulliparous	55	61.1
Multiparous	35	38.9

*GA at birth (weeks)*		
<37	5	5.6
37-38 w 6 d	30	33.3
39-40 w 6 d	51	56.7
≥41	4	4.4

*Fetal gender*		
Male	48	53.3
Female	42	46.7

*Birth weight (grams)*		
<2500	4	4.4
2500–3999	79	87.8
≥4000 g	7	7.8

*Apgar score at 5 minutes*		
<7	1	1.1
≥7	89	98.9
Total	90	100.0

**Table 2:** Measurements of longitudinal, anteroposterior, and transverse diameters and fetal thyroid lobe volume according to the side and gender of the fetus.

Measurements	Side	*n*	Mean	Median	DP	Min	Max	*P* value^*∗*^
Longitudinal	R	255	13.07	13.50	4.49	2.60	24.80	0.3935
Longitudinal	L	252	13.10	13.30	4.55	2.70	28.40	
Anteroposterior	R	255	5.45	5.40	1.95	0.90	11.10	0.0787
Anteroposterior	L	252	5.35	5.35	1.91	1.30	10.70	
Transverse	R	255	5.97	5.70	2.11	1.30	12.70	0.3530
Transverse	L	252	5.90	5.65	2.13	1.60	11.90	
Volume	D	255	289.70	227.91	253.79	2.20	1586.10	0.0789
Volume	L	251	285.92	212.36	260.09	3.27	1693.90	

Measurements^†^	Gender	n	Mean	Median	DP	Min	Max	*P* value^‡^

Longitudinal	M	138	13.41	13.80	4.20	3.85	24.35	0.1563
Longitudinal	F	117	12.65	12.70	4.59	2.65	23.65	
Anteroposterior	M	138	5.62	5.38	1.90	1.85	10.55	0.1384
Anteroposterior	F	117	5.13	5.45	1.78	1.20	9.80	
Transverse	M	138	6.16	6.00	2.05	2.00	12.30	0.0931
Transverse	F	117	5.68	5.60	2.01	1.45	9.80	
Volume	M	134	633.68	479.31	546.94	14.83	3280.10	0.0797
Volume	F	117	513.10	420.54	445.20	5.59	1955.50	

^*∗*^ANOVA for repeated measurements; ^†^mean of right/left side measurements; ^‡^Mann–Whitney Test; F: female; L: left; M: male; R: right.

**Table 3:** Equations for fitting the 10^th^, 50^th^, and 90^th^ centiles for each fetal thyroid lobe measurement according to gestational age.

*Longitudinal diameter*		
P10: −1.2000 + 0.4429 (GA)	P50: −0.3680 + 0.5120 (GA)	P90: 0.6833 + 0.5833 (GA)

*Anteroposterior diameter*		
P10: −0.2077 + 0.1615 (GA)	P50: 0.0056 + 0.2056 (GA)	P90: −0.0647 + 0.2647 (GA)

*Transverse diameter*		
P10: 0.1789 + 0.1632 (GA)	P50: 0.0136 + 0.2227 (GA)	P90: 0.0647 + 0.2882 (GA)

*Volume*		
P10: −155.95 + 11.38 (GA)	P50: −282.31 + 20.68 (GA)	P90: −457.81 + 35.98 (GA)

**Table 4:** Fitted 10th, 50th, and 90th centiles of thyroid lobe longitudinal diameter, anteroposterior diameter, and transverse diameter and volume at 14 to 40 weeks of gestation (mm and mm^3^).

Longitudinal diameter (mm)				Anteroposterior diameter (mm)				Transverse diameter (mm)				Volume (mm^3^)			
Centiles				Centiles				Centiles				Centiles			
GA	10th	50th	90th	GA	10th	50th	90th	GA	10th	50th	90th	GA	10th	50th	90th
**14**	5.00	6.80	8.85	**14**	2.05	2.88	3.64	**14**	2.46	3.13	4.10	**14**			
**15**	5.44	7.31	9.43	**15**	2.21	3.09	3.91	**15**	2.63	3.35	4.39	**15**			
**16**	5.89	7.82	10.02	**16**	2.38	3.30	4.17	**16**	2.79	3.58	4.68	**16**	20.75	21.08	29.92
**17**	6.33	8.34	10.60	**17**	2.54	3.50	4.44	**17**	2.95	3.80	4.96	**17**	32.74	44.17	71.75
**18**	6.77	8.85	11.18	**18**	2.70	3.71	4.70	**18**	3.12	4.02	5.25	**18**	44.73	67.25	113.59
**19**	7.22	9.36	11.77	**19**	2.86	3.91	4.96	**19**	3.28	4.24	5.54	**19**	56.72	90.34	155.42
**20**	7.66	9.87	12.35	**20**	3.02	4.12	5.23	**20**	3.44	4.47	5.83	**20**	68.70	113.43	197.25
**21**	8.10	10.38	12.93	**21**	3.18	4.32	5.49	**21**	3.61	4.69	6.12	**21**	80.69	136.51	239.09
**22**	8.54	10.90	13.52	**22**	3.35	4.53	5.76	**22**	3.77	4.91	6.41	**22**	92.68	159.60	280.92
**23**	8.99	11.41	14.10	**23**	3.51	4.73	6.02	**23**	3.93	5.14	6.69	**23**	104.66	182.68	322.75
**24**	9.43	11.92	14.68	**24**	3.67	4.94	6.29	**24**	4.10	5.36	6.98	**24**	116.65	205.77	364.59
**25**	9.87	12.43	15.27	**25**	3.83	5.15	6.55	**25**	4.26	5.58	7.27	**25**	128.64	228.86	406.42
**26**	10.32	12.94	15.85	**26**	3.99	5.35	6.82	**26**	4.42	5.80	7.56	**26**	140.62	251.94	448.25
**27**	10.76	13.46	16.43	**27**	4.15	5.56	7.08	**27**	4.59	6.03	7.85	**27**	152.61	275.03	490.09
**28**	11.20	13.97	17.02	**28**	4.31	5.76	7.35	**28**	4.75	6.25	8.13	**28**	164.60	298.11	531.92
**29**	11.64	14.48	17.60	**29**	4.48	5.97	7.61	**29**	4.91	6.47	8.42	**29**	176.58	321.20	573.75
**30**	12.09	14.99	18.18	**30**	4.64	6.17	7.88	**30**	5.07	6.69	8.71	**30**	188.57	344.28	615.59
**31**	12.53	15.50	18.77	**31**	4.80	6.38	8.14	**31**	5.24	6.92	9.00	**31**	200.56	367.37	657.42
**32**	12.97	16.02	19.35	**32**	4.96	6.58	8.41	**32**	5.40	7.14	9.29	**32**	212.54	390.45	699.25
**33**	13.42	16.53	19.93	**33**	5.12	6.79	8.67	**33**	5.56	7.36	9.58	**33**	224.53	413.54	741.08
**34**	13.86	17.04	20.52	**34**	5.28	7.00	8.94	**34**	5.73	7.59	9.86	**34**	236.52	436.63	782.92
**35**	14.30	17.55	21.10	**35**	5.44	7.20	9.20	**35**	5.89	7.81	10.15	**35**	248.51	459.71	824.75
**36**	14.74	18.06	21.68	**36**	5.61	7.41	9.46	**36**	6.05	8.03	10.44	**36**	260.49	482.80	866.58
**37**	15.19	18.58	22.27	**37**	5.77	7.61	9.73	**37**	6.22	8.25	10.73	**37**	272.48	505.88	908.42
**38**	15.63	19.09	22.85	**38**	5.93	7.82	9.99	**38**	6.38	8.48	11.02	**38**	284.47	528.97	950.25
**39**	16.07	19.60	23.43	**39**	6.09	8.02	10.26	**39**	6.54	8.70	11.30	**39**	296.45	552.05	992.08
**40**	16.52	20.11	24.02	**40**	6.25	8.23	10.52	**40**	6.71	8.92	11.59	**40**	308.44	575.14	1033.92

## Data Availability

The data used to support the findings of this study are available from the corresponding author upon request.

## Abbreviations

ANOVA: Analysis of variance
AP: Anteroposterior diameter
BMI: Body mass index
CRL: Crown-rump length
FGS: Fetal growth study
IRB: Institutional review board
L: Longitudinal diameter
T: Transverse diameter
Vol: Volume
WHO: World Health Organization.

## References

[1] Polak M. (2011). Thyroid disorders during pregnancy: impact on the fetus. *Hormone Research in Paediatrics*.

[2] Polak M., Luton D. (2014). Fetal thyroïdology. *Best Practice & Research Clinical Endocrinology & Metabolism*.

[3] Forhead A. J., Fowden A. L. (2014). Thyroid hormones in fetal growth and prepartum maturation. *Journal of Endocrinology*.

[4] Peleg D., Cada S., Peleg A., Ben-Ami M. (2002). The relationship between maternal serum thyroid-stimulating immunoglobulin and fetal and neonatal thyrotoxicosis. *Obstetrics & Gynecology*.

[5] Nachum Z., Rakover Y., Weiner E., Shalev E. (2003). Graves’ disease in pregnancy: prospective evaluation of a selective invasive treatment protocol. *American Journal of Obstetrics and Gynecology*.

[6] Hamada N., Momotani N., Ishikawa N. (2011). Persistent high TRAb values during pregnancy predict increased risk of neonatal hyperthyroidism following radioiodine therapy for refractory hyperthyroidism. *Endocrine Journal*.

[7] Luton D., Le Gac I., Vuillard E. (2005). Management of Graves’ disease during pregnancy: the key role of fetal thyroid gland monitoring. *The Journal of Clinical Endocrinology & Metabolism*.

[8] Thorpe-Beeston J. G., Nicolaides K. H. (1993). Fetal thyroid function. *Fetal Diagnosis and Therapy*.

[9] Agrawal P., Ogilvy-Stuart A., Lees C. (2002). Intrauterine diagnosis and management of congenital goitrous hypothyroidism. *Ultrasound in Obstetrics and Gynecology*.

[10] Morine M., Takeda T., Minekawa R. (2002). Antenatal diagnosis and treatment of a case of fetal goitrous hypothyroidism associated with high-output cardiac failure. *Ultrasound in Obstetrics and Gynecology*.

[11] Tongsong T., Wanapirak C., Kunavikatikul C., Sirirchotiyakul S., Piyamongkol W., Chanprapaph P. (2001). Fetal loss rate associated with cordocentesis at midgestation. *American Journal of Obstetrics and Gynecology*.

[12] Liao C., Wei J., Li Q., Li L., Li J., Li D. (2006). Efficacy and safety of cordocentesis for prenatal diagnosis. *International Journal of Gynecology & Obstetrics*.

[13] Cohen O., Pinhas-Hamiel O., Sivan E., Dolitski M., Lipitz S., Achiron R. (2003). Serialin utero ultrasonographic measurements of the fetal thyroid: a new complementary tool in the management of maternal hyperthyroidism in pregnancy. *Prenatal Diagnosis*.

[14] Bromley B., Frigoletto F. D., Cramer D., Osathanondh R., Benacerraf B. R. (1992). The fetal thyroid: normal and abnormal sonographic measurements. *Journal of Ultrasound in Medicine*.

[15] Ho S. S. Y., Metreweli C. (1998). Normal fetal thyroid volume. *Ultrasound in Obstetrics and Gynecology*.

[16] Achiron R., Rotstein Z., Lipitz S., Karasik A., Seidman D. S. (1998). The development of the foetal thyroid: in utero ultrasonographic measurements. *Clinical Endocrinology*.

[17] Ranzini A. C., Ananth C. V., Smulian J. C., Kung M., Limbachia A., Vintzileos A. M. (2001). Ultrasonography of the fetal thyroid: nomograms based on biparietal diameter and gestational age. *Journal of Ultrasound in Medicine*.

[18] Radaelli T., Cetin I., Zamperini P., Ferrazzi E., Pardi G. (2002). Intrauterine growth of normal thyroid. *Gynecological Endocrinology*.

[19] Bernardes L. S., Ruano R., Sapienza A. D., Maganha C. A., Zugaib M. (2008). Nomograms of fetal thyroid measurements estimated by 2-dimensional sonography. *Journal of Clinical Ultrasound*.

[20] Gietka-Czernel M., Dębska M., Kretowicz P., Dębski R., Zgliczyński W. (2012). Fetal thyroid in two-dimensional ultrasonography: nomograms according to gestational age and biparietal diameter. *European Journal of Obstetrics & Gynecology and Reproductive Biology*.

[21] Abu-Khudir R., Larrivée-Vanier S., Wasserman J. D., Deladoëy J. (2017). Disorders of thyroid morphogenesis. *Best Practice & Research Clinical Endocrinology & Metabolism*.

[22] Merialdi M., Widmer M., Gülmezoglu A. M. (2014). WHO multicentre study for the development of growth standards from fetal life to childhood: the fetal component. *BMC Pregnancy Childbirth*.

[23] Wei Y., Pere A., Koenker R., He X. (2006). Quantile regression methods for reference growth charts. *Statistics in Medicine*.

[24] Zimmermann M. B. (2009). Iodine deficiency in pregnancy and the effects of maternal iodine supplementation on the offspring: a review. *The American Journal of Clinical Nutrition*.

[25] Pearce E. N., Lazarus J. H., Moreno-Reyes R., Zimmermann M. B. (2016). Consequences of iodine deficiency and excess in pregnant women: an overview of current knowns and unknowns. *The American Journal of Clinical Nutrition*.

[26] Ozguner G., Sulak O. (2014). Size and location of thyroid gland in the fetal period. *Surgical and Radiologic Anatomy*.

